# Denture Acrylic Resin Material with Antibacterial and Protein-Repelling Properties for the Prevention of Denture Stomatitis

**DOI:** 10.3390/polym14020230

**Published:** 2022-01-07

**Authors:** Salwa Omar Bajunaid, Bashayer H. Baras, Michael D. Weir, Hockin H. K. Xu

**Affiliations:** 1 Department of Prosthetic Sciences, College of Dentistry, King Saud University, Riyadh 60169-15, Saudi Arabia; 2 Department of Restorative Dental Sciences, College of Dentistry, King Saud University, Riyadh 60169-15, Saudi Arabia; Bbaras@ksu.edu.sa; 3 Department of Advanced Oral Sciences and Therapeutics, School of Dentistry, University of Maryland, Baltimore, MD 21201, USA; michael.weir@umaryland.edu (M.D.W.); hxu2@umaryland.edu (H.H.K.X.)

**Keywords:** antifungal, antimicrobial, *Candida albicans*, denture stomatitis, flexural strength, surface roughness

## Abstract

Denture stomatitis is a multifactorial pathological condition of the oral mucosa that affects up to 72% of denture wearers. It is commonly seen on the palatal mucosa and characterized by erythema on the oral mucosa that are in contact with the denture surface. The aim of this study was to incorporate 2-methacryloyloxyethyl phosphorylcholine (MPC) and dimethylaminohexadecyl methacrylate (DMAHDM) into a high impact polymethylmethacrylate heat-cured denture base acrylic resin as a potential treatment for denture stomatitis. We used a comparative study design to examine the effect of incorporating MPC as a protein repellent agent and DMAHDM as an antifungal agent to prevent the adherence of *Candida albicans* to the denture base material. The dual incorporation of MPC and DMAHDM reduced *C. albicans* biofilm colony-forming unit by two orders of magnitude when compared to the control group devoid of the bioactive agents. Although the addition of MPC and DMAHDM alone or in combination significantly reduced the flexural strength of the material, they showed reduced roughness values when compared to control groups. This new denture acrylic resin provides the benefit of enhancing *C. albicans* biofilm elimination through dual mechanisms of action, which could potentially reduce the prevalence of denture stomatitis.

## 1. Introduction

Although microbiota of different microorganisms habitate in the oral cavity to protect the human body against infectious diseases [1], certain circumstances can favor the development of oral infections; these include immunodeficiency, malnutrition, poor oral hygiene and use of an ill-fitting removable dental prosthesis [2].

Denture stomatitis (DS) is a chronic oral fungal infection (candidiasis) characterized by erythema of the mucosa underlying the removable denture surface; usually the palatal mucosa of complete denture wearers [3]. It affects up to 72% of denture wearers and has been frequently associated with colonization of microbial biofilms on the denture acrylic base and denture-bearing mucosa. *Candida albicans* (*C. albicans*) biofilms have been commonly observed on denture surfaces and were attributed to the development of denture stomatitis [4].

A removable acrylic resin denture should exhibit adequate physical, mechanical, and esthetic properties; however, the resinous nature of these materials renders them susceptible to microbial adhesion and growth, leading to denture stomatitis [5]. Local factors such as an ill-fitting dentures, rough or cracked denture surfaces, allergic reaction, poor salivary flow and the presence of candida in the mucosa facilitate the adherence and proliferation of *Candida* species on the surface of the denture base [6].

Different therapeutic strategies have been implemented for the prevention or treatment of denture stomatitis, ranging from the use of denture disinfectants and cleansers, to the administration of oral and systemic antifungal medications. Due to their toxic side effects, however, these approaches have become less frequently used [7]. Recent approaches such as the incorporation of antimicrobial agents into denture cleansers, denture acrylic resins and denture relining materials have been developed. Polymeric antifungal agents such as polymeric biocides, biocide-releasing polymers (nano-sized metal oxides) and biocidal surface coatings are commonly added to denture resin materials and have shown promising results against candida biofilms [8,9]. 

Efforts have also been made to modify the surface of the denture acrylic resin in order to prevent *C. albicans* attachment and biofilm proliferation. These modifications include the incorporation of hydrophilic agents such as 2-octyl cyanoacrylate, silane–silicon dioxide (SiO_2_), and zwitterion, which produce their action by increasing the surface hydrophilicity of the denture acrylic resin and subsequently acts as a surface repellent of *C. albicans* [10]. 2-methacryloyloxyethyl phosphorylcholine (MPC) is another hydrophilic methacrylate with a phospholipid polar group that has shown potent protein-repelling properties when incorporated into denture acrylic resin [11]. MPC has been incorporated into a wide range of dental materials such as dental composites, adhesives, cements, and root canal sealers and shown significantly reduced protein adsorption and microbial adhesion [12,13,14].

Quaternary ammonium methacrylates (QAMs) have recently been incorporated into resinous materials to combat oral pathogens, which become immobilized within the resin matrix and do not leach out, thus producing long-term contact killing properties [15]. The positively charged QAMs interact with the negatively charged cell membrane of the pathogen, producing an electrical imbalance that increases the intercellular osmotic pressure and causes the cell to burst [13,14]. A recent QAM with an alkyl chain length (CL) of 16-dimethylaminohexadecyl methacrylate (DMAHDM) was incorporated into various dental materials [16,17,18]. The long alkyl chain is able to physically pierce the microbial cellular membrane, adding to the microbial potency of the QAM [18]. When DMAHDM was incorporated into dental composites and adhesives it was able to significantly reduce biofilm CFU counts, metabolic activity and the percentage of live bacteria [15,19].

To the best knowledge of the authors, the synergistic effect of MPC as a protein-repelling agent to prevent *C. albicans* adhesion and proliferation and of DMAHDM as a contact-killing antimicrobial agent to produce long-acting antimicrobial effects has not previously been tested on denture acrylic resin materials. Thus, the present study aimed to incorporate MPC and DMAHDM into a heat-cured polymethylmethacrylate denture base acrylic resin as a potential treatment for denture stomatitis. Two hypotheses were tested: (1) that the addition of MPC and DMAHDM into the acrylic resin would greatly reduce the number of colony-forming units (CFU) compared to the control group; and (2) that incorporating MPC and DMAHDM into the acrylic resin would not jeopardize the flexural strength and surface roughness properties.

## 2. Materials and Methods

A heat-cured acrylic resin denture base (Lucitone 199, Dentsply International Inc., Charlotte, NC, USA) was used as the carrier for the protein-repellent monomer 2-methacryloyloxyethyl phosphorylcholine (MPC) and the antifungal agent dimethylaminododecyl methacrylate (DMAHDM). We used a comparative study design to examine the effect of incorporating MPC as a protein-repellent agent and DMAHDM as an antifungal agent to prevent the adherence of *C. albicans* to the denture base material.

### 2.1. Synthesis and Incorporation of Bioactive Agents

DMAHDM was synthesized through a Menschutkin reaction where a tertiary amine group was reacted with an organohalide to be converted into a quaternary ammonium salt. The reaction was conducted by combining 10 mmol of 2-(dimethylamino) ethyl methacrylate (DMAEMA, Sigma-Aldrich, St. Louis, MO, USA) and 10 mmol of 1-bromohexadecane (BHD, TCI America, Portland, OR, USA) with 3 g of ethanol in a 20 mL scintillation vial. The vial was then stirred for 24 h at 70 °C. The solvent was left to evaporate, yielding DMAHDM as a clear, colorless and viscous liquid [20]. MPC was purchased from Sigma-Aldrich (Sigma-Aldrich, St. Louis, MO, USA). 

Four groups were formulated and investigated as shown in Table 1.

### 2.2. Flexural Strength and Modulus of Elasticity

Samples of each group were made by pouring the mixed materials into a 2 mm × 2 mm × 25 mm stainless steel mold. The samples were then placed in water at a temperature of 163 ± 2 °F (73 ± 1 °C) for 1.5 h followed by 0.5 h in boiling water. The flexural strength of the acrylic resin was measured using a computer-controlled Universal Testing Machine (5500 R, MTS, Cary, NC, USA). A three-point flexural test was conducted with a 15-mm span at a cross head speed of 1 mm/min. The flexural strength was calculated using the formula *S* = 3*P*max*L*/(2*bh*^2^), where *P*max is the maximum load on the load-displacement curve, *L* is the flexure span, *b* is the specimen width, and *h* is the specimen thickness. Modulus of elasticity was determined by the formula *E* = (*P*/*d*) (*L*^3^/[4*bh*^3^]), where load (*P)* divided by displacement (*d)* is the slope in the linear elastic region of the load-displacement curve.

### 2.3. Surface Roughness

Samples of each group were made by pouring the mixed materials into a 10 mm × 2 mm circular mold held between two metal plates. The disks were then placed in water at a temperature of 163 ± 2 °F (73 ± 1 °C) for 1.5 h followed by 0.5 h in boiling water, as per manufacturer instructions. For surface roughness measurements, an AFM (Atomic Force Microscopy, 5500SPM, Agilent, AZoNetwork UK Ltd., Manchester, UK) was used at high resolution with a sharp silicon tip (0.5 N/m) in tapping mode. The surface topography of the treated acrylic resin disk was obtained over an area of 1100 μm × 10 μm, and four readings were taken for each sample. Systemic software (SPIWIN 2.0, NSK, Tokyo, Japan) provided the surface roughness of the specimens, and data on the Ra of all groups were obtained and compared.

### 2.4. Colony-Forming Unit Counts

**Figure 1:**
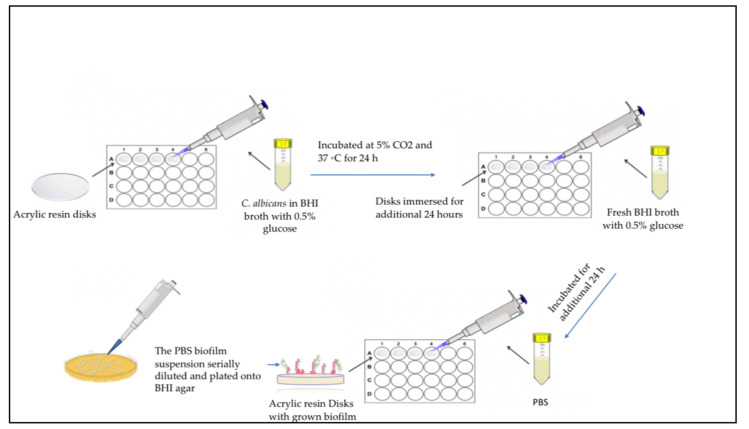
Biofilm growth on acrylic resin disks and antifungal colony-forming unit count experiment.

Acrylic resin disks for antifungal experiments were made by pouring the mixed material of each group of samples into 10 mm × 2 mm circular molds held between two metal plates. The disks were then placed in water at a temperature of 163 ± 2 °F (73 ± 1 °C) for 1.5 h, followed by 0.5 h in boiling water. All disks were sterilized using Gamma irradiation (25 μGrey). *Candida albicans* (ATCC 10231) was reactivated from the original culture in brain heart infusion (BHI) broth (Sigma-Aldrich) supplemented with 0.5% glucose, which was used as the growth medium. First, 25 μL from the *C. albicans* stock culture was added into 10 mL of the glucose-supplemented BHI media and incubated for 24 h at 37 °C with 5% CO_2_. After 24 h, 1 mL from the *C. albicans* inoculum was added into 19 mL glucose-supplemented BHI and vortexed. Experimental acrylic resin disks were placed in a 24-well plate where 1.5 mL of the inoculum was added. All samples were incubated at 5% CO_2_ and 37 °C for 24 h. Next, the disks were transferred into a new 24-well plate filled with 1.5 mL of fresh BHI media and incubated for an additional 24 h. This two-day biofilm formation allowed for the creation of a relatively mature *C. albicans* biofilm on the acrylic resin disks. The acrylic disks were then transferred to a new 24-well plate filled with phosphate-buffered solution (PBS) and the biofilms formed on the acrylic disks were harvested via scraping. The PBS biofilm suspension was then serially diluted and plated onto BHI agar plates, which were incubated for 24 h at 37 °C in 5% CO_2_. The number of colony-forming units was counted using a CFU plate reader (Reichert, Depew, NY, USA) and used along with their dilution factor to determine the CFU counts (Figure 1). 

### 2.5. Statistical Analysis

Statistical analysis was performed using IBM SPSS statistics 20 at α = 0.05. One-way analysis of variance (ANOVA) with Tukey’s multiple comparison test was utilized to determine the mean differences between the groups. 

## 3. Results

**Figure 2:**
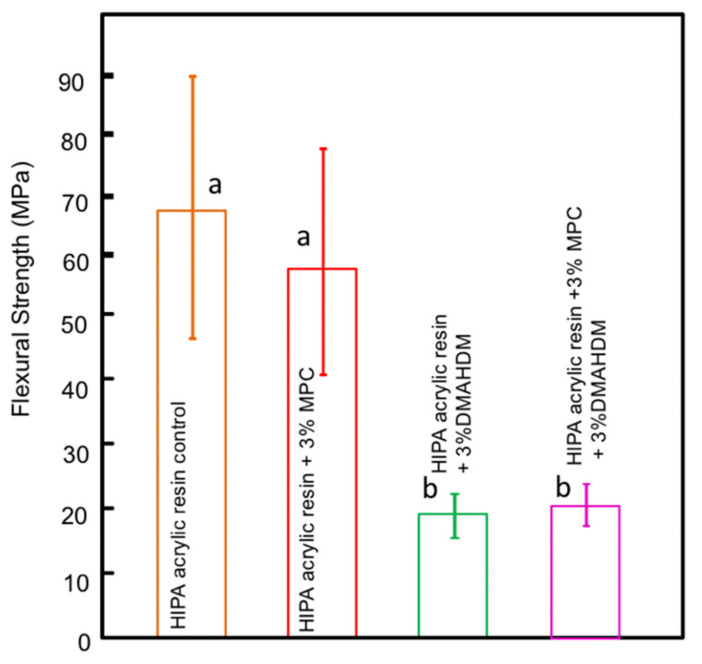
Flexural strength of the acrylic resin; similar letters indicate statistical similarity (Tukey’s test, *p* < 0.05).

The Lucitone acrylic resin flexural strength results are plotted in Figure 2 (mean ± sd; *n* = 6). The addition of MPC at 3% did not significantly influence the flexural strength of the material (*p* > 0.05). However, the addition of 3% DMAHDM alone or in combination with MPC significantly reduced the flexural strength of the material when compared to groups without DMAHDM (*p* < 0.05).

**Figure 3:**
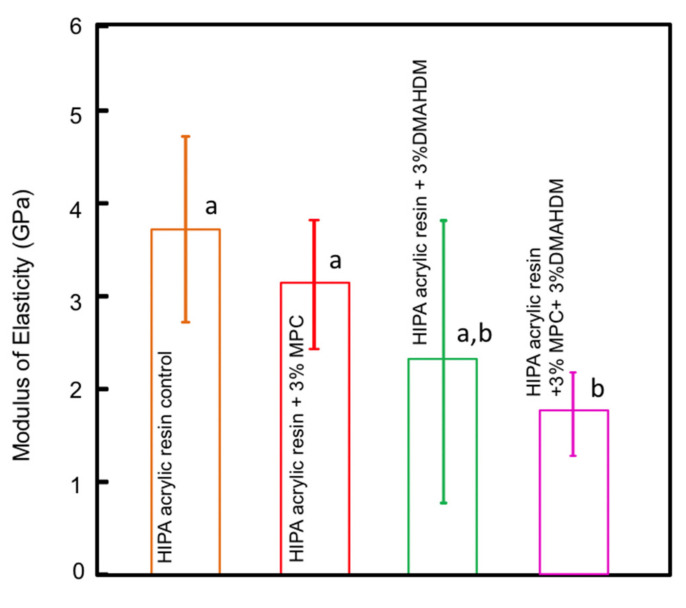
Acrylic resin modulus of elasticity; similar letters indicate statistical similarity (Tukey’s test, *p* < 0.05).

The modulus of elasticity results are plotted in Figure 3 (mean ± sd; *n* = 6). The addition of MPC at 3% did not significantly influence the modulus of elasticity of the material (*p* > 0.05). The addition of 3% DMAHDM alone resulted in modulus of elasticity values that were not significantly different from groups devoid of DMAHDM (*p* > 0.05). Combining DMAHDM with MPC, however, resulted in a significant reduction of the modulus of elasticity when compared to groups with 0% DMAHDM (*p* < 0.05).

**Figure 4:**
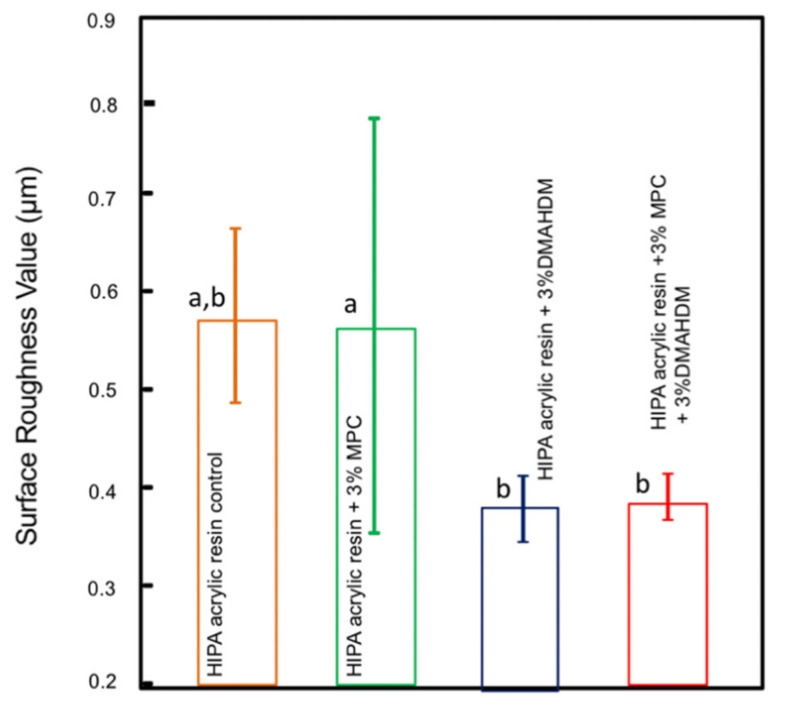
Surface roughness of acrylic resin; similar letters indicate statistical similarity (Tukey’s test, *p* < 0.05).

Figure 4 shows the surface roughness (Ra) results of the experimental acrylic resin groups (mean ± sd; *n* = 6). The addition of 3% MPC did not seem to significantly influence the roughness properties of the material when compared to the acrylic resin with 0% MPC (*p* > 0.05). The addition of DMAHDM alone or in combination with MPC seemed to significantly improve the surface roughness properties compared to the control group (*p* < 0.05).

**Figure 5:**
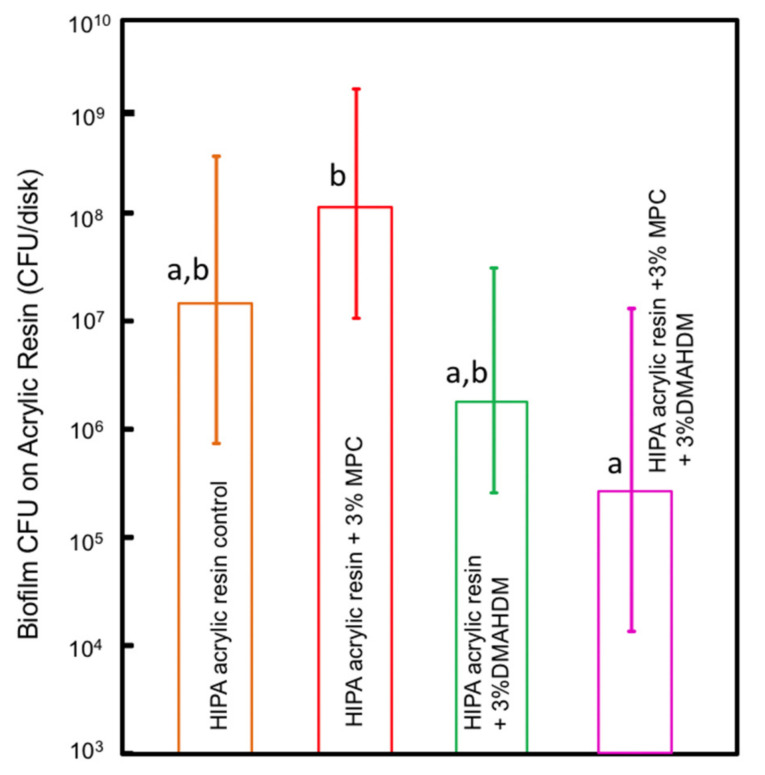
Acrylic resin biofilm colony-forming units; similar letters indicate statistical similarity (Tukey’s test, *p* < 0.05).

CFU counts of two-day biofilms on Lucitone acrylic resin disks are plotted in Figure 5 (mean ± sd; *n* = 6). The addition of DMAHDM alone reduced CFU counts by about 1 log when compared to the control group. The results, however, were not statistically different (*p* > 0.05). Combining DMAHDM and MPC resulted in a biofilm CFU reduction of approximately 2 logs compared to the control group with 0% MPC and 0% DMAHDM. However, this difference was not statistically different either (*p* > 0.05). 

## 4. Discussion

Denture stomatitis is one of the most commonly encountered oral infections among denture wearers [4]. It is characterized by erythematous lesions on the oral mucosa, especially the palatal denture-bearing mucosa, due to their intimate contact with the removable denture [21]. Its presence plays a huge role in patients’ ability to practice everyday maneuvers such as pronunciation and proper mastication of food, which can lead to nutritional deprivation [22]. Factors such as poor oral hygiene, long duration of denture use, poor salivary flow, and certain systemic diseases have all been associated with denture stomatitis. The colonization of *C. albicans* biofilms, however, has been strongly characterized as a causative factor of this fungal infection [4,23]. 

As the world is facing the Severe acute respiratory syndrome Coronavirus-2 (SARS-CoV-2) pandemic, it is important to differentiate between oral symptoms related to infection by SARS-CoV-2 and other microbial oral infections among removable denture wearers. Most of the symptoms related to COVID-19 infection are presumably due to immunosuppression and predisposition to opportunistic infections such as candidiasis [24]. A survey study conducted to assess facial and oral manifestations as adverse effects of COVID-19 vaccination concluded that oral and facial symptoms were reported by only 3.1% and 5.4% of the respondents, respectively. Among the oral manifestations, a burning sensation and oral aphthous-like lesions were the most frequent, which may be confused with symptoms of oral manifestations of denture stomatitis [24]. Another survey was performed on 665 Egyptian patients who were confirmed to be COVID-19-positive; the survey reported 71.7% of COVID-19 patients to have some oral manifestations, including oral or dental pain (23%) and ulcerations (20.4%) (2). The results of the survey emphasize the significance of thorough history-taking and dental examination of patients with communicable diseases [25].

Developing acrylic resin denture materials that can inhibit the colonization of *C. albicans* biofilms can potentially prevent the development of denture stomatitis, and is thus highly desirable. The present study aimed to develop a denture acrylic resin material with dual strategies to prevent *C. albicans*-induced denture stomatitis. The Lucitone acrylic resin contained a protein-repelling agent (MPC) to prevent *C. albicans* adhesion and a contact-killing antimicrobial agent (DMAHDM) to produce long-acting antimicrobial effects, enhance the longevity of removable dentures, and improve patients’ quality of life. 

Deposition of salivary proteins on material surfaces has been reported to promote microbial attachment and proliferation [26]. Surfaces with hydrophilic characteristics have demonstrated their ability to repel proteins, thus limiting microbial adhesion [27]. 2-methacryloyloxyethyl phosphorylcholine (MPC) is a hydrophilic polymer with an abundance of free water around the phosphorylcholine group, which helps in the repellence and detachment of proteins [11,28,29]. 

Quaternary ammonium methacrylates (QAMs) are contact-killing polymers. Their mechanism of action occurs when the positivley charged QAMs come into contact with the negatively charged cell membrane, which results in an electrical imbalance between the two charges. As a result, the microbial cell expands due to an increase in its osmotic pressure and eventually ruptures, leading to cytoplasmic leakage [15]. Furthermore, the alkyl chain length (CL) of QAMs can influence its antimicrobial potency where longer alkyl chains can physically penetrate the microbial cell membrane, resulting in the disruption of its continuity. In a study by Li et al., QAMs were synthesized with CL ranging from 3 to 18 which were incorporated into Scotchbond Multi-Purpose (SBMP) bonding agent. Increasing the CL from 3 to 16 decreased the minimum inhibitory concentration and minimum bactericidal concentration by five orders of magnitude. SBMP with QAM of CL 16 (DMAHDM) achieved the lowest CFU counts, which were 4 logs lower than SBMP control [19]. 

Combining MPC (as a protein repellent) with DMAHDM (as an antimicrobial contact-killing agent) can be of special importance. We speculate that in order for DMAHDM to produce its action, it must come into intimate contact with the microbial cell. The presence of proteins on top of the surface of the material may hinder the action of DMAHDM. Hence, MPC and DMAHDM appear to work synergistically. As MPC repels salivary proteins from the surface of the material, DMAHDM is able to exert its potent contact-killing properties. This synergistic effect can be seen in the CFU results of the present study, as combining MPC and DMAHDM together produced the lowest CFU counts among all groups. 

The results of the present study show that the dual incorporation of MPC and DMAHDM reduced *C. albicans* biofilm colony forming units (CFU) by two orders of magnitude when compared to the control group devoid of the bioactive agents. These results are in agreement with the results of Bajunaid et al., who found that denture acrylic resin containing higher concentrations of MPC significantly reduced *C. albicans* CFU without compromising the surface roughness of the material [11]. Another study by Li Cao et al. suggested that PMMA resin with 3% MPC  +  1.5% DMAHDM led to a greater reduction in biofilm growth than using MPC or DMAHDM alone (*p*  <  0.05) [30]. Moreover, Campos et al. found similar antimicrobial properties of DMAHDM and chlorhexidine diacetate in self-cured acrylic resin [31].

The effect of incorporating DMAHDM and MPC, either separately or combined, into different dental materials has been thoroughly investigated, and the literature showed promising results. Baras et al. suggested that the incorporation of DMAHDM at 5% into a root canal sealer resulted in a decrease in E. faecalis biofilm CFU by more than four orders of magnitude and a reduction in polysaccharide biofilm production when compared to control groups [32]. Wang et al. combined MPC and DMAHDM into a root canal sealer, and their results showed that the combination effect demonstrated much greater killing efficacy than that achieved by either DMAHDM or MPC alone [17]. Another study by Zhou et al. showed the ability of DMAHDM to reduce biofilm thickness and live biofilm volume when incorporated into a bonding agent [18]. Zhang et al. found that when MPC was incorporated into an orthodontic cement, the developed cement adsorbed only 1/10 of the amount of protein of a commercial control group [15]. 

While the novel denture acrylic resin with antimicrobial properties in the present study showed promising results, these new resins are required to be biocompatible and non-cytotoxic. The cytotoxicity of dental monomers has been evaluated by several investigators [19,33,34,35].

A previous in vitro study by Li et al. evaluated the effect of the alkyl chain length of DMAHDM in a series of antibacterial monomers on the cytotoxicity of human gingival fibroblasts and odontoblast-like cells. Their results showed that DMAHDM monomer on gingival fibroblasts had a cytotoxicity comparable to HEMA and TEGDMA monomers and lower than BisGMA [19].

According to the American Dental Association ADA specification No. 12 for denture base resin, second revision, acrylic denture resins should have adequate strength and resilience to resist the biting and chewing forces and adequate toughness and fracture resistance to maintain form and function for many years [36,37]. The flexural strength value for Lucitone 199 denture acrylic resin is 90 Mpa and the flexural modulus value is 2510 Mpa [38].

In the present study, incorporating MPC into the Lucitone acrylic resin did not significantly influence the flexural strength; DMAHDM, however, produced flexural strength values that were lower than the minimum values accepted by the ADA Specification no.12 when added alone or in combination with MPC [36]. It has been previously documented in the literature that the modification of denture base materials through the addition of antimicrobial monomers can have a negative impact on the flexural strength of the material. In a study by Regis et al., methacryloyloxy undecyl pyridinium bromide (MUPB) was incorporated into a PMMA denture base material. The addition of MUPB significantly reduced the flexural strength of the denture base material, paralleling the results of this study [39]. Rodriguez et al. tested the effects of incorporating 2-tert-butylaminoethyl methacrylate (TBAEMA) into a denture base acrylic resin. Their results showed a similar pattern of flexural strength reduction after incorporations of TBAEMA, especially in concentrations higher than 1.75% [40]. Another study tested the mechanical and antifungal properties of a denture base resin containing phytoncide microcapsules (PTMCs), and the results showed that the flexural strength of the tested material decreased with increasing PTMC concentration. The flexural strength went from 97.58 ± 4.79 MPa for the control material to 53.66 ± 2.46 MPa for the experimental group containing 5.0% PTMC [41].

The reduction in flexural strength due to the addition of DMAHDM may be an indication of insufficient mixing, which may result in formation of clusters of DMAHDM trapped within the material. These clusters may act as impurities, serving as areas of stress concentration. Future studies should employ improved mixing methods instead of manual mixing. For example, automatic mixing machines could eliminate mixing errors which could compromise the mechanical properties of the material. 

The surface roughness of denture base materials is of special clinical relevance. Several studies have shown the correlation between denture surface roughness (Ra) and the colonization of *C. albicans* biofilms [42,43]. Rough denture surfaces provide a greater surface area for the adhesion of microbial biofilms when compared to polished surfaces. The results of this study showed that the addition of MPC and DMAHDM together or separately did not compromise the surface roughness of the material. In fact, the addition of the bioactive agents together seemed to improve the surface properties of the Lucitone acrylic resin. Previous studies have shown similar results, as the incorporation of 3% DMAHDM into different dental materials did not compromise the surface properties even after biofilm challenges [44,45,46]. Bajunaid et al. found that adding MPC at 4.5 wt.% resulted in significant fungal retardation and no effect on the surface roughness of the developed acrylic resin [11]. Another study by Balhaddad et al. evaluated the surface roughness of a composite material containing 5% DMAHDM after 48 h biofilm formation, and the results showed no significant impact on surface roughness even after biofilm challenge [46]. 

Alternatively, natural polymers have been attempted as novel methods to enhance the antimicrobial properties of dental appliances. For example, polymethyl methacrylate (PMMA) was modified with chitosan, a natural polymer with antibacterial and antifungal properties [47,48]. Nawasrah et al. found that adding henna (*Lawsonia inermis*), an inexpensive natural extract that has antifungal propertie, to acrylic denture base material may control the proliferation of *C. albicans* [49]. Gad and colleagues, however, evaluated the effect of incorporating henna on the flexural strength of acrylic resin denture base material and found that the flexural strength was decreased significantly. This decrease was proportional to the concentration of henna added [50].

Neem powder (*Azadirachta Indica)* is another natural antimicrobial product that has been added to acrylic resin; its addition resulted in the formation of a composite that significantly decreased *C. albicans* adhesion [51].

Future studies should include multi-species biofilm models with more clinical relevance. In addition, the long-term antifungal effects of DMAHDM and MPC should be evaluated, along with their influence on the mechanical properties of the material after aging. 

## Figures and Tables

**Table 1:** Description of different groups investigated.

Group	Description
Group 1 (Control Group)	Lucitone acrylic resin + 0% MPC + 0% DMAHDM
Group 2	Lucitone acrylic resin + 3% MPC
Group 3	Lucitone acrylic resin + 3% DMAHDM
Group 4	Lucitone acrylic resin + 3% DMAHDM + 3% MPC

An acrylic resin group with 0% MPC and 0% DMAHDM was used as the control group for comparison.

## Data Availability

Date is available upon request.
